# Rare findings of indirect inguinal hernia repair during laparoscopic PIRS technique in children: a two-center audit

**DOI:** 10.3389/fped.2026.1742114

**Published:** 2026-02-23

**Authors:** Kutay Bahadir, Sylwester Gerus, Sumeyye Sozduyar, Ufuk Ates, Gulnur Gollu, Meltem Kologlu, Aydin Yagmurlu, Murat Cakmak, Dariusz Patkowski, Ergun Ergun

**Affiliations:** 1 Department of Pediatric Surgery, Ankara University School of Medicine, Ankara, Türkiye; 2 Department of Pediatric Surgery and Urology, Wroclaw University Faculty of Medicine, Wrocław, Poland; 3 Department of Pediatric Surgery and Urology, Ege University School of Medicine, Izmir, Türkiye

**Keywords:** Direct hernia, Femoral hernia, inguinal hernia, Laparoscopy, Spigelian hernia

## Abstract

**Introduction:**

This study aimed to present rare findings encountered during laparoscopic inguinal hernia repair in children.

**Methods:**

The study included children who underwent laparoscopic surgery for inguinal hernia (IH). The focus was on unexpected intraoperative findings other than a typical inguinal hernia. These included direct and femoral hernias, various hernia variants, and other unusual presentations.

**Results:**

A total of 790 children were included in the study. Unexpected laparoscopic findings were observed in 22 cases. The mean age of the patients was 33 months (range: 1–168 months), and the mean weight was 12.5 kg (range: 2–46 kg). Direct hernias were identified in seven children. Four children had an incarcerated appendix within the hernia sac, one of whom required an appendectomy. Omental incarceration was noted in three children, and no hernia was found in two cases. Additionally, single cases of femoral hernia, Spigelian hernia, pantaloon hernia, intraabdominal spermatic cord cyst, suspicious gonadal structure, and incarcerated uterus with ovary was observed. Direct inguinal hernia was identified in seven children; four were repaired laparoscopically, whereas three underwent open repair. No intraoperative or postoperative complications were reported.

**Conclusions:**

The advantages provided by the laparoscopic approach include comprehensive visualization of the intraabdominal cavity allowing for prompt identification and safer management of rare or atypical findings.

## Introduction

Inguinal hernia repair is one of the most commonly performed surgical procedures by pediatric surgeons (1–3). The primary cause of inguinal hernias in children is a patent processus vaginalis (PPV), which typically results in the development of an indirect inguinal hernia (IIH) (4). In contrast, direct inguinal hernia (DIH) and femoral hernia are rare entities in the pediatric population, with reported incidences ranging from 0.2% to 2% and 0.2% to 1%, respectively (5, 6). These conditions most commonly present as recurrent hernias following primary open repairs (6). However, with the increasing adoption of laparoscopic techniques, their true incidence may be higher than previously estimated (7).

Rarer anomalies, such as hernia en pantaloon, have also been documented (8). Additionally, although IIH is often the initial clinical suspicion, laparoscopic exploration can uncover unexpected findings such as incarcerated omental tissue, herniated ovaries, fallopian tubes, uterus, or even an inflamed appendix due to strangulation (2, 7, 9). Given their rarity, these conditions may present diagnostic and therapeutic challenges for pediatric surgeons.

Randomized evidence summarized in a recent systematic review and meta-analysis has indicated that laparoscopic pediatric inguinal hernia repair is associated with a shorter operative duration, whereas recurrence and overall complication rates are comparable with those of open repair (10). In addition, laparoscopy facilitates inspection of the contralateral internal ring and may help identify contralateral pathology without the need for re-sedation (10). Despite the described advantages of laparoscopy, it has been noted that consensus regarding the superiority of laparoscopic versus open pediatric inguinal hernia repair remains limited, and the debate on optimal treatment strategies continues (11).

The aim of this study is to report rare intraoperative findings and describe their management during laparoscopic inguinal hernia repair using the Percutaneous Internal Ring Suturing (PIRS) technique at two high-volume pediatric surgery centers.

## Materials and methods

### Study design and population

This retrospective two-center audit was conducted at two high-volume tertiary pediatric surgery centers between 2016 and 2021. All children who underwent laparoscopic inguinal hernia repair during the study period were included in serial order. Patients who underwent primary open inguinal hernia repair, those with previous inguinal surgery, and cases with known associated congenital or systemic conditions were excluded from the analysis. Patients with unusual intraoperative findings identified during laparoscopy constituted the study focus. The study was conducted in accordance with the Declaration of Helsinki and approved by the local ethics committee. Data were collected from the Departments of Pediatric Surgery and Pediatric Urology at Ankara University Faculty of Medicine (Türkiye) and Wroclaw University Faculty of Medicine (Poland).

All procedures were performed or directly supervised by consultant pediatric surgeons with near-equal experience in laparoscopic inguinal hernia repair, ensuring standardization of surgical expertise across both centers.

### Definition of unusual findings

The study focused on intraoperative findings that deviated from typical IIH presentations, excluding cases with known associated conditions. Prior to data extraction, an “unusual finding” was predefined as any intraoperative laparoscopic diagnosis other than a typical indirect inguinal hernia associated with a patent processus vaginalis (PPV). Unusual findings were categorized as: (i) non-indirect hernia types (e.g., direct inguinal hernia, femoral hernia); (ii) rare hernia variants (e.g., pantaloon hernia, Spigelian hernia); (iii) unexpected hernia sac contents or incarceration (e.g., Amyand's hernia, omental incarceration, ovarian/uterine incarceration); (iv) other unexpected intraabdominal/inguinal pathology relevant to the presentation or operative management (e.g., intraabdominal spermatic cord cyst, suspicious gonadal structure); and (v) laparoscopic hernia nonvizualization, defined as a closed internal ring with no PPV and no peritoneal sac/defect.

### Data collection

**Figure 1:**
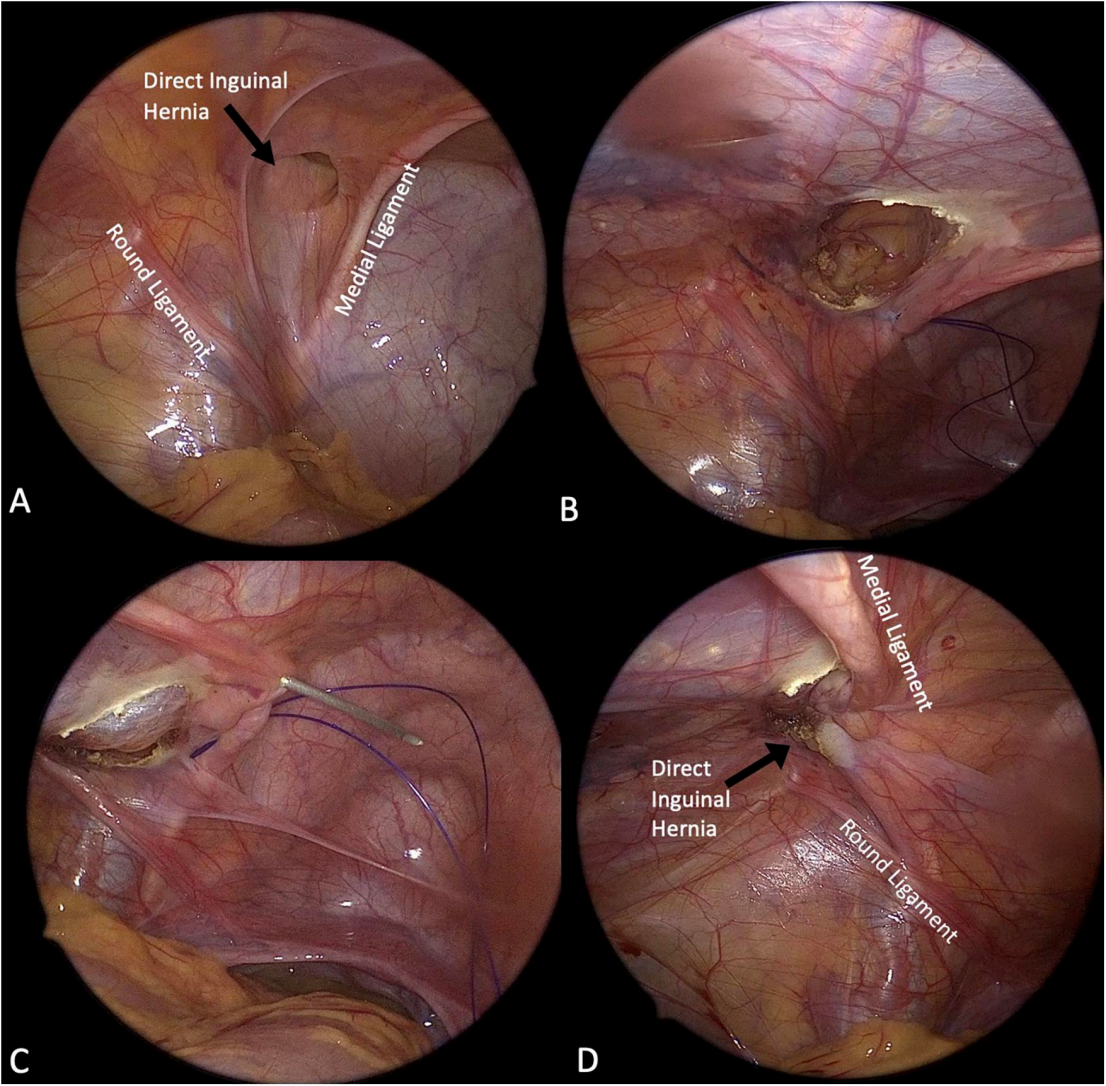
Laparoscopic management of direct inguinal hernia using a modified percutaneous suturing technique (modified PIRS). **(A)** Laparoscopic view demonstrating a direct inguinal hernia defect in relation to the round ligament and the medial umbilical ligament. **(B)** First needle pass: under direct laparoscopic visualization, an 18-G needle loaded with a 2/0 non-absorbable suture is introduced percutaneously and the suture is advanced along the lateral margin of the direct defect. **(C)** Second needle pass: the suture is then advanced along the medial margin of the defect, completing the modified percutaneous closure; an extracorporeal knot is tied to approximate and close the defect. **(D)** Final laparoscopic appearance showing complete closure of the direct defect; when deemed necessary, the medial umbilical ligament may be advanced over the repair for additional reinforcement.

In most cases, the diagnosis of inguinal hernia was based on clinical symptoms and physical examination. Preoperative diagnosis was not based solely on a positive silk-glove sign. Surgery was indicated when a visible or palpable intermittent groin swelling was reported by the family and/or demonstrated on examination, typically accentuated during crying, coughing, or straining, and consistent with a reducible inguinal hernia. Patients were selected for laparoscopic repair based on a preoperative clinical diagnosis of inguinal hernia and institutional practice during the study period. Laparoscopic PIRS repair was the preferred approach in both centers in patients who were candidates for laparoscopic surgery and pneumoperitoneum induction. Patients with contraindications to laparoscopy who underwent primary open inguinal hernia repair were excluded from this study. Procedures were routinely recorded, and all available recordings were retrospectively reviewed by two consultant pediatric surgeons to confirm and categorize unusual intraoperative findings. A standardized data-extraction approach was used to document the type of finding, laterality, need for additional trocars, and intraoperative management. Discrepancies were resolved by discussion and consensus. A 5-mm camera trocar was inserted into the peritoneal cavity via an umbilical incision created using the open (Hasson) technique. Pneumoperitoneum was established with CO₂ insufflation according to the patient's age and body habitus. A 30-degree laparoscope was used to inspect both internal inguinal rings and assess for contralateral pathology. When a patent processus vaginalis (PPV)/open internal ring was identified, the patient was positioned in Trendelenburg with contralateral tilt to facilitate exposure by allowing the bowel to fall away from the operative field. Percutaneous access was then obtained using an 18-G needle. For PIRS repair, a 2/0 non-absorbable suture was placed circumferentially around the internal ring under direct laparoscopic visualization, and an extracorporeal knot was tied to achieve complete ring closure while avoiding entrapment of the vas deferens and gonadal vessels. Modified percutaneous repair for DIH (modified PIRS): When a direct inguinal hernia was identified intraoperatively (medial to the inferior epigastric vessels), a modified percutaneous suturing approach was performed under laparoscopic visualization. An 18-G needle loaded with a 2/0 non-absorbable suture was introduced percutaneously, and the medial defect was closed by two sequential needle passes—one from the lateral margin and one from the medial margin—so that the suture encompassed the defect and approximated the tissues overlying the medial weakness without entrapping the vas deferens or gonadal vessels. The knot was tied extracorporeally under direct vision to achieve defect closure. When deemed necessary, the medial umbilical ligament was mobilized and advanced over the defect and secured as reinforcement during closure (Figure 1). The contralateral side was assessed routinely and repaired when indicated. Contralateral findings were classified intraoperatively as either contralateral “open canal/true hernia” or contralateral PPV. To minimize subjective interpretation, contralateral findings were categorized using predefined visual criteria under standard pneumoperitoneum and 30° inspection. A contralateral “open canal/true hernia” was recorded when a well-formed hernia-type peritoneal sac was seen extending beyond the internal ring into the inguinal canal, typically appearing as a deep funnel-shaped sac configuration with a clearly gaping canal (i.e., a configuration consistent with a hernia sac rather than a simple patency). In contrast, contralateral PPV only was defined as patency of the internal ring/processus vaginalis without a formed hernia sac configuration, characterized by a shallow peritoneal dimple or slit-like opening at the ring without a funnel-shaped sac descending into the canal and without herniated contents. When available, the classification was corroborated on retrospective video review by two consultant pediatric surgeons and any discrepancies were resolved by consensus. Contralateral “open canal/true hernia” was identified and was repaired during the same anesthesia using PIRS. Contralateral PPV was defined as a patent processus vaginalis/patent internal ring without a clear hernia configuration, and these cases were managed by observation. For the purpose of this study, contralateral pathology was recorded as an asymptomatic contralateral PPV/open internal ring identified via laparoscopy (i.e., visible patency of the internal ring with a peritoneal sac/communication). Because these findings may represent an occult predisposition rather than a clinically apparent hernia, they are referred to as contralateral PPV/open internal ring throughout the manuscript. Additional working trocars were inserted selectively when required, for safe reduction and assessment of incarcerated contents (e.g., Amyand's hernia, omental incarceration, or ovarian incarceration), after which PIRS closure was completed. The same operative workflow was followed in both centers. DIH was defined intraoperatively as a medial defect relative to the inferior epigastric vessels. During the early phase of the study period, when DIH was unexpectedly encountered during laparoscopy, three patients were managed with classical open repair in accordance with the institutional practice at that time. After completion of the learning curve, subsequent DIH cases were managed laparoscopically using a modified percutaneous suturing technique to close the medial defect under direct visualization, with extracorporeal knot tying. In this study, “no hernia” refers to patients with preoperative clinical findings suggestive of inguinal hernia, but with no hernia identified during laparoscopy, defined as the absence of a patent processus vaginalis (PPV) with a closed internal inguinal ring and no peritoneal sac/defect on inspection.

The collected data included demographics, hernia laterality, surgical technique, unusual findings, intraoperative and postoperative complications, and patient outcomes. Internal ring diameter was intraoperatively assessed via laparoscopic inspection; however, ring diameter was not recorded as a standardized quantitative variable in this retrospective dataset.

Postoperative follow-up was performed according to routine institutional practice in both centers. All patients were assessed clinically before discharge and were routinely scheduled for an early outpatient visit (typically within 7–10 days). Longer-term follow-up beyond the early postoperative period was not standardized in this retrospective dataset and depended on local referral pathways and family preference; therefore, recurrence could not be evaluated reliably, and age-stratified follow-up analyses were not performed.

### Statistical analysis

This study was designed as a descriptive retrospective case series focusing on rare intraoperative findings. Accordingly, the data were presented as counts and proportions for categorical variables and mean (range) for continuous variables. Given the very low frequency of individual unusual findings (several entities occurred only once), subgroup or comparative inferential analyses were not pre-specified and would be underpowered; therefore, no hypothesis-testing subgroup analyses were performed.

## Results

Among the 790 patients, 22 cases (2.8%) presented with unusual laparoscopic findings. Patient ages ranged from 1 to 168 months, with a mean of 33 months, and the mean body weight was 12.5 kg (range: 2–46 kg). Routine laparoscopic inspection identified contralateral findings in 234/790 (29.6%) patients. Of these, 161/234 were classified as contralateral open canal/true hernia and were repaired during the same procedure, whereas 73/234 were classified as PPV only and were managed by observation. During 1-year follow-up, no new symptoms suggestive of contralateral hernia were reported in the observation group.

### Unusual intraoperative findings

**Figure 2:**
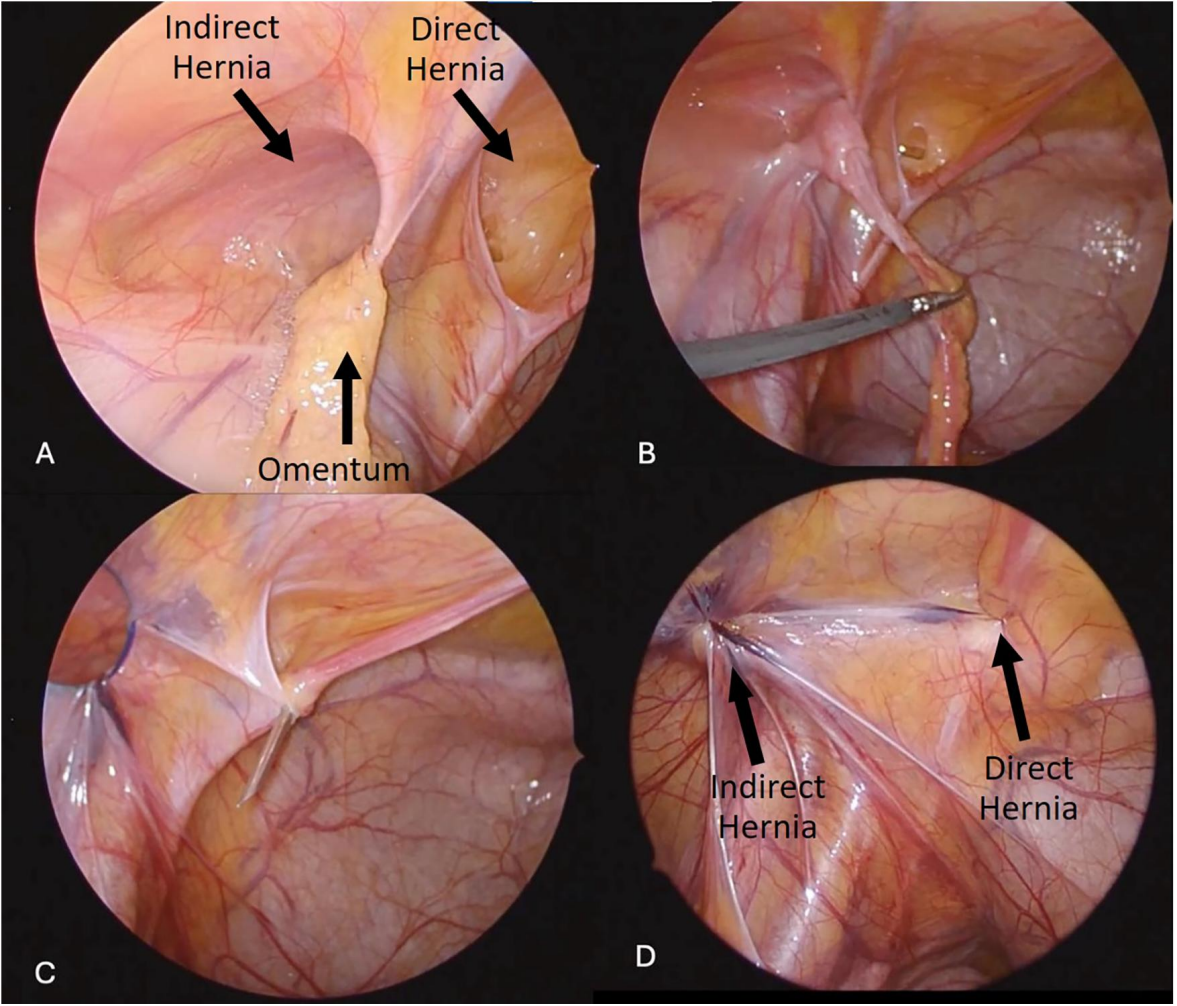
Laparoscopic findings and stepwise repair of coexisting direct and indirect inguinal hernia with omental involvement. **(A)** Laparoscopic view demonstrating concurrent direct and indirect inguinal hernia components with omentum herniating through the defect. **(B)** Reduction and dissection of the omentum from the internal ring region to enable safe repair. **(C)** Percutaneous passage of a non-absorbable suture loop via an 18-G needle under laparoscopic guidance to achieve defect closure. **(D)** Final laparoscopic appearance showing complete closure of both the direct and indirect components after repair.

**Figure 3:**
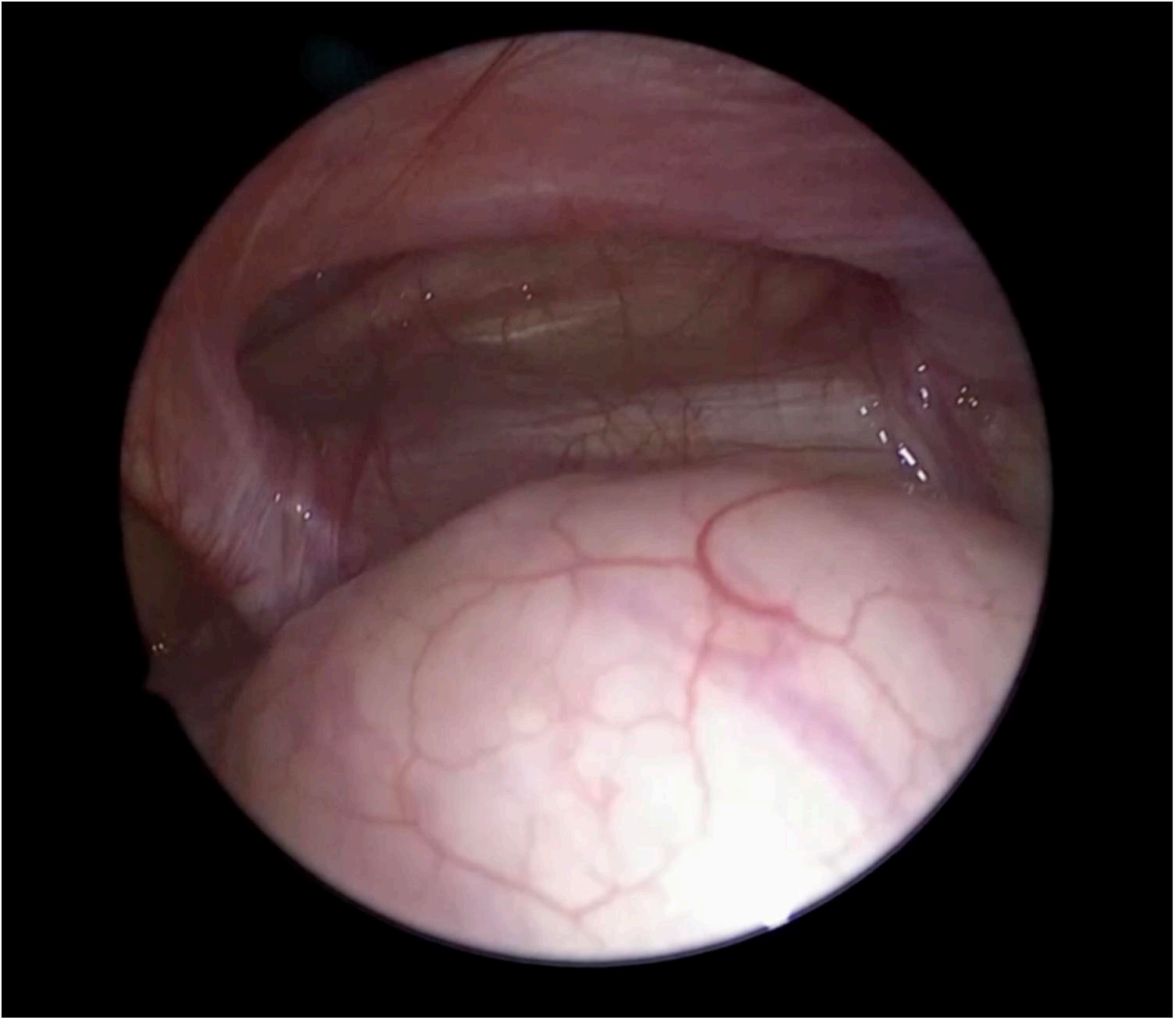
Laparoscopic view of Spigelian hernia demonstrating the lateral abdominal wall defect prior to repair.

Unusual findings included DIH in seven children, an incarcerated appendix within the hernia sac in four (one requiring appendectomy), omental incarceration in three, and hernia nonvisualization in two cases (Figure 2). In both patients, surgery had been indicated based on a history of intermittent groin swelling reported by the family and/or demonstrated on examination during crying/coughing/straining; however, laparoscopy showed a closed internal ring with no PPV, consistent with the absence of an inguinal hernia at the time of surgery. Additionally, one case each of femoral hernia, Spigelian hernia, hernia en pantaloon, intraabdominal spermatic cord cyst, incarcerated uterus with ovary, and suspicious gonadal structures were identified (Table 1). The pantaloon hernia was confirmed laparoscopically by the presence of combined medial and lateral components and was repaired using the PIRS technique with internal ring closure under direct vision. For the Spigelian hernia, a pre-repair laparoscopic image was available and included, however, a post-repair image could not be obtained due to technical failure of the video recording system (Figure 3).

**Table 1:** Number of unusual findings.

Finding	*n*
Total number of inguinal hernia	790
Contralateral PPV/open internal ring	234
Total number of unusual findings	22
Direct hernia	7
Incarcerated appendix	4 (one patient required appendectomy)
Omental incarceration	3
No inguinal hernia	2
Femoral hernia	1
Spigelian hernia	1
Pantaloon hernia	1
Intraabdominal spermatic cord cyst	1
Incarcerated ovarian and uterus	1
Suspicious gonadal structure	1

Routine laparoscopic inspection of the contralateral side was performed in all patients. Contralateral PPV/open internal ring (occult contralateral finding) was identified in 234 of 790 patients (29.6%).

### Operative management and outcomes

Direct inguinal hernia (DIH) was identified in seven children; four were managed laparoscopically using modified PIRS, whereas three underwent classical open repair. All patients were discharged on the day of surgery, and no intraoperative or postoperative complications were reported. No age- or ring-diameter–specific activity restrictions were prescribed postoperatively; children were allowed to return to normal activities as tolerated, with routine advice to avoid strenuous exertion during the early postoperative period.

## Discussion

Inguinal hernia repair remains one of the most frequently performed day-case surgical procedures by pediatric surgeons (12). While indirect inguinal hernia (IIH) is the most common form in children, other types such as direct inguinal hernia (DIH), femoral hernia, and various rare anomalies may also be encountered intraoperatively (7). These atypical findings can pose diagnostic and therapeutic challenges and may increase the risk of complications or necessitate additional interventions. The present study aimed to highlight such unexpected findings during laparoscopic PIRS procedures at two high-volume pediatric surgery centers and to describe their management strategies.

Diagnosing DIH and femoral hernias during open surgery in children, particularly at the time of primary repair, is relatively uncommon (5, 7, 13, 14). In many cases, persistent or recurrent symptoms after surgery lead to further imaging studies, which may reveal previously undiagnosed or overlooked hernias. Laparoscopy allows direct visualization of the internal inguinal anatomy and facilitates identification of contralateral or atypical findings during the same procedure. While this may reduce the likelihood of missed diagnoses and the need for additional surgery in some patients, re-operation rates were not assessed in the present study. In line with this concept, increased use of laparoscopy has revealed that the incidence of such rare hernias may be higher than previously reported (7). Our findings align with contemporary evidence provided by randomized studies showing that laparoscopic repair offers operative advantages without clear differences in recurrence or overall complication rates compared with open repair (10).

DIH accounts for approximately 2%–3% of all pediatric inguinal hernias and is characterized by its medial location relative to the inferior epigastric vessels and the inguinal ligament (14). Esposito et al. demonstrated that laparoscopic DIH repair involves excision of the associated lipoma, closure of the defect with non-absorbable sutures, and reinforcement using the vesical ligament (15). Femoral hernias, though rare (1%–2% of pediatric hernias), are similarly difficult to detect. These hernias occur as a protrusion through the femoral ring, located inferior to the inguinal ligament and medial to the femoral vessels (13, 14).

Amyand's hernia—defined as the presence of the appendix within the inguinal sac—is another rare but clinically significant condition. Its most serious complication is appendiceal incarceration with resultant appendicitis (16). Diagnosis is typically made intraoperatively. In our series, Amyand's hernia was identified in four patients, and one required appendectomy in addition to standard hernia repair.

Ovarian herniation is a relatively common finding, present in approximately 15%–20% of female pediatric inguinal hernia cases. However, herniation of the uterus is exceedingly rare. The etiology remains unclear, although it is hypothesized to be related to laxity of the suspensory ligament of the ovary. Uterine herniation may also be associated with congenital anomalies such as Müllerian aplasia and Mayer-Rokitansky-Küster-Hauser (MRKH) syndrome (4)

Laparoscopic inguinal hernia repair offers several advantages, including the ability to visualize and diagnose contralateral hernias, identify combined or atypical hernias, and detect additional intraabdominal pathology (7). In our series, the utilization of laparoscopy facilitated the identification of rare hernia variants such as pantaloon hernia and spigelian hernia thereby allowing definitive treatment in the same setting using the PIRS technique. This comprehensive visualization enables prompt diagnosis and management, potentially reducing the need for additional surgical interventions (7).

Although many laparoscopic techniques require additional working trocars, the PIRS technique generally employs only a single camera port. In our study, the PIRS method was successfully used to repair all unusual hernia types, except in eight cases—four involving Amyand's hernia, three with omental incarceration, and one with ovarian incarceration—where additional trocars were required to safely complete the procedure. Notably, three of the DIH cases were not managed laparoscopically in our series; these patients underwent classical open repair and therefore were not counted as requiring additional trocars.

This study has several limitations. Primarily, as a retrospective analysis, causal inference and generalizability is limited. In addition, although the overall cohort was large, the number of rare intraoperative entities was small, which limited subgroup analyses and statistical power for comparative assessments. Unavailability of some intraoperative images of the hernia variants, as well as the post-repair images of the Spigelian hernia as a result of technical failure of the video recording system. Another limitation of this study was that the internal ring size was not measured and recorded systematically; therefore, outcomes could not be analyzed according to ring diameter or age subgroups. Re-operation rates and long-term outcomes could not be evaluated; therefore, no causal inference can be made regarding a reduction in re-operations. In addition, contralateral PPV/open internal ring identified during laparoscopy does not necessarily mean progression to a clinically apparent hernia; therefore, the natural history of these asymptomatic findings and the rate of future metachronous hernia could not be determined from our dataset. Contralateral classification relied on predefined qualitative laparoscopic morphology rather than measured ring diameter or sac length. Long-term follow-up was not standardized, precluding reliable assessment of recurrence and any correlation of recurrence with age groups, particularly in older children. Although no symptoms developed in the observed PPV group during 1-year follow-up, progression beyond this period cannot be excluded, and longer-term natural history could not be established in this retrospective dataset.

In conclusion, laparoscopic surgery provides superior anatomical visualization compared to the open approach and facilitates the identification of rare or unexpected findings that might otherwise go unnoticed. This not only supports the implementation of a more appropriate surgical strategy but may help reduce the likelihood of missed diagnoses and the need for additional procedures; however, re-operation rates were not assessed in this study. A comprehensive understanding of these rare presentations is essential for pediatric surgeons to ensure optimal intraoperative decision-making and patient outcomes.

## Data Availability

The raw data supporting the conclusions of this article will be made available by the authors, without undue reservation.
